# Hfinger: Malware HTTP Request Fingerprinting

**DOI:** 10.3390/e23050507

**Published:** 2021-04-23

**Authors:** Piotr Białczak, Wojciech Mazurczyk

**Affiliations:** 1 CERT Polska/Research and Academic Computer Network (NASK), Kolska 12, 01-045 Warsaw, Poland; 2 Institute of Computer Science, Warsaw University of Technology, Nowowiejska 15/19, 00-665 Warsaw, Poland; wojciech.mazurczyk@pw.edu.pl

**Keywords:** fingerprinting, malware analysis, malicious network traffic analysis, HTTP protocol analysis, pcap file analysis, malware tracking, malware identification

## Abstract

Malicious software utilizes HTTP protocol for communication purposes, creating network traffic that is hard to identify as it blends into the traffic generated by benign applications. To this aim, fingerprinting tools have been developed to help track and identify such traffic by providing a short representation of malicious HTTP requests. However, currently existing tools do not analyze all information included in the HTTP message or analyze it insufficiently. To address these issues, we propose Hfinger, a novel malware HTTP request fingerprinting tool. It extracts information from the parts of the request such as URI, protocol information, headers, and payload, providing a concise request representation that preserves the extracted information in a form interpretable by a human analyst. For the developed solution, we have performed an extensive experimental evaluation using real-world data sets and we also compared Hfinger with the most related and popular existing tools such as FATT, Mercury, and p0f. The conducted effectiveness analysis reveals that on average only 1.85% of requests fingerprinted by Hfinger collide between malware families, what is 8–34 times lower than existing tools. Moreover, unlike these tools, in default mode, Hfinger does not introduce collisions between malware and benign applications and achieves it by increasing the number of fingerprints by at most 3 times. As a result, Hfinger can effectively track and hunt malware by providing more unique fingerprints than other standard tools.

## 1. Introduction

Currently, malicious software (malware) developers use Hypertext Transfer Protocol (HTTP) as one of the primary carriers for malicious communication. According to Miller et al. [1], HTTP is the most common protocol used in the Command and Control (C&C) traffic, more popular than Hypertext Transfer Protocol Secure (HTTPS). It is utilized by malware, e.g., to connect to the C&C server to register or obtain commands, check the infected machine’s IP address, or download additional modules. Moreover, it can be used to perform DDoS (Distributed Denial of Service) attacks or to click on referral links, thus creating revenue.

To identify and discern different malware communication activities, network traffic fingerprinting methods can be applied. The notions of a fingerprint and fingerprinting as the act of creating a fingerprint are similar to the notions of classic forensic work, where the fingerprint is an impression of human fingers’ friction ridges. In the field of computer science, a working definition of a fingerprint is a short representation of a larger object [2]. The most crucial property of fingerprinting is that two different objects have different fingerprints, and the probability of a collision, i.e., an event when two different objects have the same fingerprint, is low. File fingerprinting is one of the application examples, where cryptographic hash functions, e.g., such as SHA-256, are used to create identification tags for the fingerprinted files.

However, it is not only files that can be fingerprinted. Network traffic can also be used for this purpose. Various network protocols can be analyzed to represent the exchanged data, which then, in turn, can be used for identification purposes. The process of fingerprinting can be conducted in an active or passive mode. The former is performed with a modification of the standard network traffic, for example, by sending carefully crafted messages. In the latter, no changes are introduced, and network traffic is only monitored. The most popular practical usage examples are passive Operating System fingerprinting (as realized, e.g., in p0f https://lcamtuf.coredump.cx/p0f3, accessed on 26 March 2021), web browser fingerprinting (like in, e.g., privacy research and advocacy service at https://panopticlick.eff.org/, accessed on 26 March 2021) or network service discovery (as performed by, e.g., Nmap https://nmap.org/, accessed on 26 March 2021). Network protocols can also be fingerprinted to identify, track, or detect malware. Until now, several such examples of network traffic fingerprinting methods and tools exist, and they are discussed in detail in Section 2.

From the variety of network protocols, HTTP fingerprinting is a promising approach to provide identification and tracking of malware communications, which is crucial for malware analysts in their daily work while defending networks. Currently, several tools have been proposed that help fingerprinting HTTP traffic, such as FATT (https://github.com/0x4D31/fatt, accessed on 26 March 2021), p0f (https://lcamtuf.coredump.cx/p0f3, accessed on 26 March 2021), or Mercury (https://github.com/cisco/mercury, accessed on 26 March 2021). However, they all share the same limitation. In our opinion, they do not analyze all information included in the HTTP messages or analyze it insufficiently. For example, the above-mentioned tools do not analyze the payload of the request, and the URI analysis is at most limited to value encoding. Note that both these features have already been proven to be useful for malware detection and identification purposes, for example, as described by Li et al. [3] or Perdisci et al. [4]. Fingerprints created with such an approach omit information that can potentially identify and discern various malware families’ requests.

To address these issues, we propose the Hfinger tool that aims to fingerprint malware HTTP requests more comprehensively. In more detail, Hfinger processes HTTP requests and generates a fingerprint based on the URI, protocol version, the request method, headers and their values, and the request’s payload. The tool’s main goal is to produce unique representations of malware HTTP requests, thus providing a mechanism for the identification of such requests in network traffic of various applications. The fingerprint created by Hfinger represents a malware request in a short and concise form that can still be interpretable by a human analyst. Hfinger was designed to be used with exact match searching mechanisms, which provide means for direct fingerprint searching without using wildcard techniques. Exact match searching is supported by, for example, many security monitoring and logging tools. In this vein, it is intended to provide similar search functionality as malware sample hashes such as SHA-256. Nevertheless, its overt nature can still help analysts by giving basic information about the request or by finding patterns in the network traffic.

Hfinger does not provide direct identification of particular malware families or directly detect malware per se. However, it can identify requests that can be labeled as malicious using other information sources, such as Intrusion Detection Systems. The tool can also be used in threat hunting to uncover unknown requests that were omitted by other security solutions but that share fingerprint with those identified as malicious. The tool is open source and has been published at https://github.com/CERT-Polska/hfinger, accessed on 26 March 2021. The research presented in this paper is focused on HTTP requests of Windows-based malware. The utilization of only HTTP requests is related to the fact that the server responses may be unavailable in some of the fingerprinting tool usage scenarios. For example, when analyzing an old malware sample for which C&C servers no longer work, or server responses are unreliable because the malicious infrastructure was sinkholed and the received messages are different from the original. From this perspective, using only requests for fingerprinting purposes can be more reliable in analyzing malware’s actual behavior. Moreover, focusing on Windows-based malware is related to the fact that it is still the most frequently attacked platform despite the constant increase in the number of threats on mobile platforms. According to AV-TEST Institute in 2019, more than 75% of malware targeted Windows operating system [5].

To prove the effectiveness of the proposed Hfinger tool we have conducted an extensive experimental study. To this aim, we based the performed evaluation on four main metrics that measure fingerprint collision probability for various malware families (separately and including benign software), the number of created fingerprints, and their entropy. Moreover, to determine the performance of the proposed solution for malware HTTP request fingerprinting, we use real-world malware and benign applications data sets containing HTTP traffic. In more detail, the former consists of 121 popular malware families represented using 401,566 HTTP requests, while the latter incorporates 248,657 HTTP requests generated by popular Windows applications, including web browsers. Additionally, the effectiveness of the developed tool has been compared with the three existing, previously mentioned community-proven HTTP fingerprinting tools, i.e., FATT, p0f, and Mercury.

Considering the above, the main contributions of this paper are as follows:

- Proposing Hfinger—a new malware HTTP request fingerprinting tool;
- Performing a review and analysis of popular HTTP fingerprinting tools;
- Providing an extensive experimental evaluation of the proposed approach and its comparison with the popular, existing HTTP fingerprinting tools.

The rest of the paper is structured as follows. First, Section 2 describes the most notable related work. Then, in Section 3, we present the proposed HTTP fingerprinting tool. Next, Section 4 contains details on the chosen experimental methodology, while in Section 5 obtained results are included and discussed. Section 6 showcases potential practical usage scenarios for Hfinger and pinpoints its main limitations. Finally, Section 7 concludes our work and indicates potential future research directions.

## 2. Related Work

In this section, first we review the most important work related to the topic of this paper and compare it to Hfinger. Then we describe existing popular tools used for HTTP traffic fingerprinting and we discuss their limitations. Finally, we compare them to the solution proposed in this paper.

An important distinction between the presented research solutions and tools must be drawn. The former were created to provide an extensive research analysis of a particular problem which, in some cases, resulted in creation of a tool or a system that solves the stated problem. On the other hand, the latter were primarily focused on creation of a tool that solves a specific technical (rather than a research) problem and the tool’s analysis is typically quite limited.

### 2.1. Proposed Research Solutions

Research on web browser fingerprinting is directly related to HTTP malware fingerprinting, and this topic has been extensively covered in the literature (cf. Laperdrix et al. [6]). This type of fingerprinting is based on active and passive techniques in which information about different features of the environment, web browser, and OS are extracted. While for active fingerprinting different techniques are used, such as JavaScript to query information about the canvas, a list of browser plugins, or screen resolution, passive fingerprinting techniques analyze requests sent by web browsers. Common techniques involve checking the values of popular headers such as *User-Agent*, *Accept*, or *Content-Encoding* but also headers’ order. Hfinger utilizes these passive fingerprinting techniques; however, they are extended, for example, with URI and payload analysis.

Fingerprinting of HTTP network traffic can be used to create models of applications present in a monitored network and used as a baseline for detecting unknown applications that can be malicious. Bortolameotti et al. presented in [7] DECANTeR a system for detection of HTTP network traffic that is anomalous for analyzed host. It passively extracts fingerprints of benign applications running on the host. This process involves extracting information from clustered *POST* and *GET* requests in the form of *Host* header value, constant header fields, average request size, *User-Agent* header value, *Accept-Language* header value, and the size of outgoing information in the cluster.

Bortolameotti et al. presented in [8] a system for the detection of anomalous traffic. Their system uses two models of header-value entropy and header sequence extracted from multiple requests to create known applications’ fingerprints. After the training phase, the system can evaluate if an unknown request is similar to already known applications or it originates from a new application. Comparing these two approaches to Hfinger shows they were designed with different objectives than the latter. Their goal is to provide a model of application behavior based on multiple HTTP requests to create a baseline for detecting outlying applications in a particular network, thus providing malware detection. On the other hand, Hfinger is focused on the unique representation of malware HTTP requests, providing a fingerprint for each separate request. Such an approach allows analyzing network traffic without the baseline model creation stage and analyzing network traffic with single requests, for example, when the infrastructure of analyzed malware is not working anymore. Furthermore, while all these systems analyze similar parts of the requests, Hfinger utilizes a broader set of features for fingerprint generation and analyzes all requests, regardless of their method. In contrast, for example, DECANTeR fingerprints clustered *GET* and *POST* requests only.

Various approaches have been proposed for fingerprinting other popular network protocols. Many studies focus on HTTPS protocol, where the primary research objectives are HTTPS network traffic presence identification or identification of services utilizing HTTPS for communication (cf. [9]). SMTP network traffic fingerprinting can be used to identify malware families as presented in [10,11]. SMTP messages, SMTP extensions, and IMF fields are used to create different e-mail clients’ dialects, thus providing a method for their identification. DNS protocol fingerprinting can be used as a method to detect DNS amplification DDoS attacks [12], identify DNS servers [13], or for the detection of bots [14]. Segal et al. in [15] presented a white paper on fingerprinting of HTTP/2 protocol clients. When used for malware network traffic fingerprinting, the presented approaches can be applied to identify malware families but also to detect some specific operations, such as sending spam messages or performing DDoS attacks.

Other approaches for network traffic fingerprinting with more generic methods exist, too. For example, Holland et al. in [16] proposed nPrint—a system for standard representation of network traffic. For every analyzed packet, its representation is created that maps all packet bytes to a feature vector representing all possible headers of a particular protocol. The authors claim that their system can generate data suitable as input for machine learning algorithms in classification problems. Unfortunately, when fingerprinting HTTP network traffic, nPrint needs to be configured with appropriate rules, defining which parts of the messages should be extracted. Therefore, it does not provide ready-made methods for HTTP fingerprinting.

Table 1 introduces the classification of the existing fingerprinting research based on its application scenario. It also shows that these approaches utilize fingerprinting for various purposes—some provide identification of benign services or applications, while the other uncover malicious activities and software.

Although the research solutions described above provide fingerprinting mechanisms of various network protocols, they differ from Hfinger in several aspects. Browser fingerprinting methods use, mainly, active analysis techniques and are focused on one type of HTTP clients. Hfinger utilizes only passive analysis techniques, and, despite the focus on malware requests, its design and performance analysis considered the presence of benign HTTP clients. DECANTeR and HeadPrint systems utilize multiple HTTP requests to create a baseline model of the observed network to identify requests that do not fit the created model, thus detecting unknown applications. Hfinger is focused on the unique representation of single malware HTTP requests, which is a different research goal. nPrint provides generic fingerprints of various network protocols and requires the creation of configuration to produce HTTP request fingerprints. The configuration is not provided by the authors, so research into optimal HTTP representation is needed. Conversely, Hfinger is focused only on HTTP, for which a complete optimization has been performed. Segal et al. analyze HTTP in version 2 that is different than previous protocol versions in data transfer and representation techniques. Thus their analysis techniques cannot be directly compared to Hfinger. Other reviewed research solutions analyze protocols different than HTTP.

### 2.2. Existing HTTP Fingerprinting Tools

In this subsection, HTTP fingerprinting tools similar to the Hfinger are described. Three tools (FATT, Mercury, and p0f) have been selected based on capability of passive, pcap file based analysis of HTTP requests without any major code modification. Source code and documentation of these three applications are public, and they are well known in professional network security community.

Other tools fingerprinting HTTP do exist, but they use active fingerprinting for web browser identification (e.g., FingerprintJS—https://github.com/fingerprintjs/fingerprintjs, accessed on 26 March 2021) or they perform only server fingerprinting (httprecon—https://www.computec.ch/projekte/httprecon/, accessed on 26 March 2021, httprint—https://net-square.com/httprint.html, accessed on 26 March 2021, or nmap—https://nmap.org/, accessed on 26 March 2021). As such, they cannot be compared to the same extent to Hfinger as FATT, Mercury, or p0f.

When using the classification of fingerprinting research solutions presented in Table 1 the three reviewed tools, with some extensions, can be classified into groups providing identification of client applications, unknown applications, or malware families. All these tools produce fingerprints that, after the labeling process, can be used for searching the application defined by the labeled fingerprint. Conversely, if the created fingerprint does not fit a list of known applications, it can be used to identify an unknown application.

The three presented tools will be further analyzed and compared with Hfinger in Section 5. Details about their source code and configuration used in the analysis are presented in Section 4.3.

#### 2.2.1. FATT

FATT—fingerprint all the things (https://github.com/0x4D31/fatt/, accessed on 26 March 2021)—is a tool for fingerprinting protocols such as SSL/TLS, SSH, RDP, HTTP, gQUIC. For HTTP, it provides means for fingerprinting headers of requests and responses by extracting header names into a list and computing MD5 hash from it. Depending on the chosen reporting format, the tool outputs additional contextual information, such as request URI, request full URI, request method and protocol version, and *User-Agent* value. However, these components are not used as a part of the fingerprint.

#### 2.2.2. p0f

p0f—passive OS fingerprinting (https://lcamtuf.coredump.cx/p0f3/, accessed on 26 March 2021)—is a tool mainly known for its capabilities of OS fingerprinting. In version 3 of the tool, additional functionality of the HTTP fingerprinting was added. It can fingerprint client requests but also server responses. However, the request support is limited only to *GET* and *HEAD* methods, which in the authors’ opinion is a huge drawback. The request fingerprint provides information about protocol version, order-preserving list of headers present in the request, absent headers, and *User-Agent* header value. When creating a list of headers, headers defined as optional are marked with a question mark “?”, values of Host and User-Agent headers are skipped. For other headers, their values are provided, creating a list of header name and value pairs. If any of the headers *User-Agent*, *Host*, *Connection*, *Accept*, *Accept-Encoding*, *Accept-Language*, *Accept-Charset* or *Keep-Alive* is absent, such information is provided by the fingerprinter. Note that Hfinger, proposed in this paper, also provides an order-preserving list of headers, but header values are provided in a separate part of the fingerprint and only for popular headers.

p0f provides information about automatic fingerprint generation. The tool can also handle user-provided fingerprints and search in pcap files for such fingerprints. The search can identify requests even when some other headers occur between those present in the fingerprint or when some of the headers are missing—those that are marked as optional, providing that p0f can detect mismatches between the identified fingerprint and the declared *User-Agent* value. The Hfinger does not offer this functionality.

#### 2.2.3. Mercury

Mercury is a network metadata capture and analysis framework (https://github.com/cisco/mercury, accessed on 26 March 2021). It provides fingerprinting capabilities for protocols such as TLS, DTLS, SSH, HTTP, and TCP. Additionally, it can perform application identification using the created fingerprints. The HTTP fingerprinting can be performed on both requests and responses. Note that the description presented below covers only request fingerprinting.

The tool analyzes the HTTP request to extract information about request method, protocol version, and a list of present headers, if they are on a predefined list of popular headers, including *Accept-Charset*, *Accept-Language*, *Cache-Control*, *Host*, and *User-Agent*. Some of the headers are presented with their values, for example *Accept*, *Accept-Encoding*, or *Connection*. All these features are represented using their hexadecimal values, forming the actual fingerprint. Beside the fingerprint, the tool provides contextual information that presents in a clear form URI and values of headers such as: *User-Agent*, *Host*, *X-Forwarded-For*, and *Via*.

#### 2.2.4. Limitations of Current Tools

The described tools use a limited set of features for HTTP request fingerprinting, and the performed analysis is limited. FATT neglects URI, method, protocol version, payload, and headers’ values during the fingerprint generation process. p0f does not analyze URI, payload, and method of request. Mercury does not process the payload, and URI analysis is limited to simple encoding that is even not added to the fingerprint. In both p0f and Mercury, the list of popular headers included in fingerprint creation can be improved as well as the list of headers whose values are added to the fingerprint.

The described tools’ analysis is insufficient to achieve a satisfactory level of malware HTTP request fingerprinting uniqueness and can be improved. This paper will try to prove this statement by comparing the results of these three tools with the proposed approach. Moreover, to the authors’ best knowledge, there is no extensive academic study that systematically analyzes the effectiveness of FATT, p0f, or Mercury for malware HTTP fingerprinting.

## 3. Hfinger

In this section, Hfinger functioning is presented in detail, along with request features that the tool investigates. Moreover, the process of the fingerprint generation is thoroughly explained.

### 3.1. Hfinger’s Workflow

**Figure 1:**
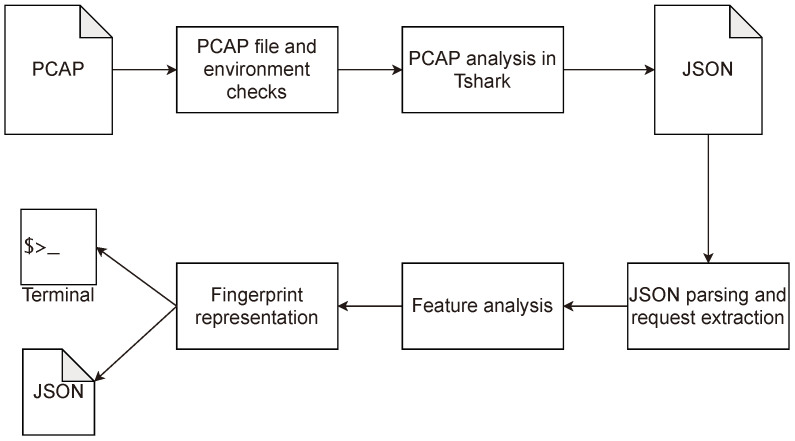
Hfinger’s data workflow.

Hfinger has been created using Python3 language and additionally it utilizes TShark (https://www.wireshark.org, accessed on 26 March 2021, minimum version 2.2.0) to reassemble TCP segments into HTTP requests. An overview of the tool’s workflow is presented in Figure 1.

The tool firstly checks the execution environment to determine whether minimal criteria for running are met (e.g., Tshark binary is present) and if the input file is a valid PCAP file. If successful, the tool calls Tshark binary and feeds the analyzed PCAP file into it. TShark is configured to output a JSON file covering only HTTP requests. Then, the output JSON file is parsed by Hfinger and the requests are extracted. In the next step, the extracted requests are analyzed to generate the feature values. In the final step the feature values are joined together with a “|” (pipe) in a particular order, forming the HTTP request fingerprint. Depending on the users’ choice, the results in the JSON format are either printed to the terminal or written to a file. The JSON output consists of the basic network information about each request: request timestamp, IP addresses, utilized ports, and the actual request fingerprint. Features analyzed by Hfinger are described in detail in the next section.

### 3.2. Analyzed Features

In this section, the features analyzed by Hfinger are presented and discussed. The chosen feature set utilized by the developed tool relies on the authors’ previous work [17], previously published research (see [18] for URI features), and the authors’ own malware analysis experience. In general, extracted features can be divided into three groups depending on the part of the request that they refer to: URI, headers, and payload.

#### 3.2.1. URI Features

These features are used to extract information from the URI part of a request. They include:

- *Length of the URI*, represented as a logarithm with base 10 of the actual URI length (provided as a floating-point number rounded to one decimal place or rounded to an integer);
- *Number of directory levels in the URI*, represented as an integer;
- *Average length of the directory*, represented as a logarithm with base 10 of the actual average length of the directory (provided as a floating-point number rounded to one decimal place or rounded to an integer);
- *Extension of the file requested in the URI*, if applicable. The extension is extracted only if it is present on a defined list of popular extensions to prevent extracting nonsensical values;
- *Length of the variable part of the URI*, where the URI parameters are stored, represented as a logarithm with base 10 of the length (provided as a floating-point number to rounded one decimal place or rounded to an integer);
- *Number of variables in the URI*, represented as an integer;
- *Average value length*, represented as a logarithm with base 10 of the actual average value length (provided as a floating-point number rounded to one decimal place or rounded to an integer).

#### 3.2.2. Header Structure Features

They provide information about headers, their values, extended with information about the request method, and HTTP version. The analyzed features consist of (in the order used in the fingerprint):

- *Request method*, presented as the first two characters of the method name;
- *HTTP version*, expressed as a single number, depending on the first digit after the dot in the protocol definition, for example, “1” for “1.1” version and “9” if no protocol version is defined;
- *Representation of header order in the analyzed request*, where the headers are expressed by the chosen encoding scheme. This scheme provides a list of popular headers for which encoding is provided to shorten the fingerprint length. However, if the header is not on the list, its name is hashed using the 32-bit Fowler–Noll–Vo hash function in version 1a (FNV1a) [19], and the hexadecimal representation of the hash is used as the name. If the header name does not begin with an upper case letter (or any first letter of the parts of a compound header name, for example, *Accept-Encoding*), an exclamation mark *!* is prepended to the header representation;
- *Representation of popular header’s values*—the following headers are analyzed to extract their value:
  - *Connection*,
  - *Accept-Encoding*,
  - *Content-Encoding*,
  - *Cache-Control*,
  - *TE*,
  - *Accept-Charset*,
  - *Content-Type*,
  - *Accept*,
  - *Accept-Language*,
  - *User-Agent*.
  If the value is present on a list of popular values, it is encoded with a chosen short encoding representation. If it is not on the list, the values are hashed using FNV1a. The representation is provided as an encoded header name and its encoded value, separated by “:” (colon), and such pairs are separated using “/” (forward slash). If the header can have multiple values, their representation is separated by “,” (comma). The order of the headers is preserved. Additionally, the value of the *User-Agent* header is always represented as the FNV1a hash.

#### 3.2.3. Payload Features

They are extracted if the payload of a request is not empty. Payload features consist of three features (in the order used in the fingerprint):

- *Presence of non-ASCII characters*, represented as a single letter “N” if non-ASCII characters are present, and “A” otherwise,
- *Shannon entropy of the payload*, represented as a floating-point number rounded to one decimal place or rounded to an integer,
- *Payload length*, represented as a logarithm with base 10 of the actual payload length (provided as a floating-point number rounded to one decimal place or rounded to an integer).

#### 3.2.4. Numerical Features’ Representation

As presented above, some of the numerical features are inherently real numbers and have to be represented as a float type. During the design phase, a decision was made to round such values to one decimal place or round them to an integer. The rounding mode can have a significant impact on Hfinger evaluation; thus, in Section 5.1 an analysis of which version of the representation should be chosen for each of these features is discussed.

### 3.3. Fingerprint Generation

**Figure 2:**
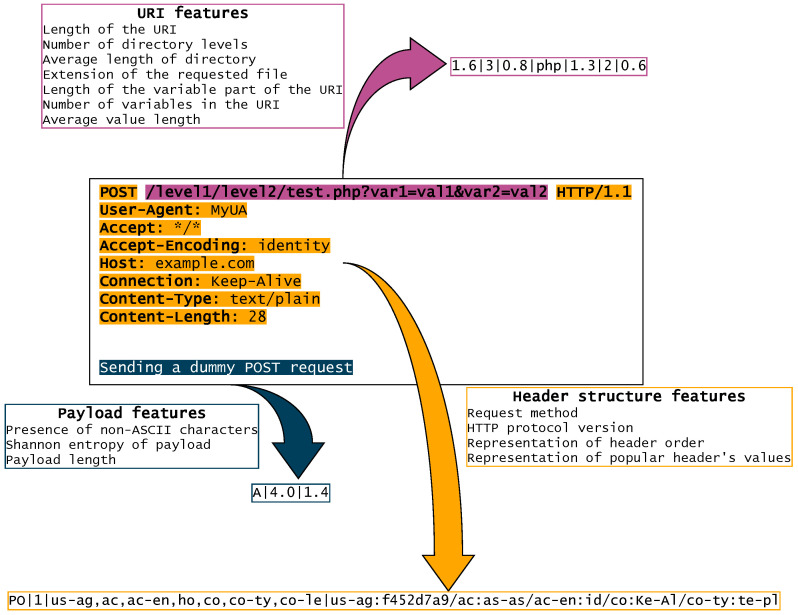
An example of a HTTP POST request fingerprint produced by Hfinger.

Features described above are used to create the HTTP request fingerprint. Figure 2 illustrates an overview of an exemplary Hfinger fingerprint generation. All analyzed features are presented, including floating-point representation, what may vary from the final feature set selection presented in Section 5.1.

As presented in Figure 2, Hfinger analyzes three parts of the HTTP request to generate a fingerprint. Firstly, the URI part is analyzed and the feature values are generated. For example, in Figure 2, the URI length is 43 characters, there are 3 directory levels, and a *PHP* file is requested. These features are represented in the generated fingerprint part as *1.6*, *3*, and *php* respectively.

Secondly, header structure features are extracted to generate the second part of the final fingerprint. For instance, using the example in Figure 2, the method is *POST*, protocol is in version *1.1*, *User-Agent* header has value of *MyUA*, and *Connection* header has value of *Keep-Alive*. These values are transformed into corresponding fingerprint parts: *PO*, *1*, *us-ag:f452d7a9*, and *co:Ke-Al*, respectively. Header names on their own and in pair with values are parts of broader structure features, representing order of all headers in the request, or representing popular header’s values, also order wise. The encoding of header names and values is provided by Hfinger’s configuration file.

The third part of the fingerprint is generated on the basis of request’s payload data. In the example in Figure 2, the request contains payload of *Sending a dummy POST request*. This string is built only from ASCII characters and is 28 characters long. The corresponding features generated by Hfinger are *A* and *1.4*.

Final fingerprint is created by combining the three generated parts in predefined manner: URI features, header structure features, and payload features. The fingerprint length is variable as it is dependent on the request’s structure and data, for example, payload features are provided only if payload data is present. The final selection of features in particular fingerprint parts and their rounding mode also affects the format and length of the fingerprint. This will be described in Section 5.1.

## 4. Experimental Methodology

In this section, we first present details related to the malware and benign application data sets that are later used during experimental evaluation. Next, we describe existing fingerprinting tools utilized while conducting comparison analysis. Finally, we outline and define performance measures and experimental methodology.

### 4.1. Malware Data Set

Malware data set is compiled from two pcap data sets: one was used in the authors’ previous work [17] and originates from CERT Polska’s sandboxing environment and Malware Capture Facility Project (https://www.stratosphereips.org/datasets-malware, accessed on 26 March 2021). The second data set was derived from a newer version of CERT Polska’s malware analysis platform.

The first data set contains 26,133 pcap files analyzed and labeled in the previous work. To this end, Snort IDS with Emerging Threats Pro (ET Pro—https://www.proofpoint.com/us/threat-insight/et-pro-ruleset, accessed on 26 March 2021) and Snort Registered (https://www.snort.org/downloads#rules, accessed on 26 March 2021) rulesets were used. More information about this data set can be found in [17].

The second pcap data set consists of 8674 files and it was created specifically for the purpose of this research. The analyzed pcap files originate from the CERT Polska’s malware analysis platform, where Windows-based malware samples are analyzed. The malware samples are obtained from various open-source feeds, for example, Abuse.ch (https://abuse.ch/, accessed on 26 March 2021), from external user uploads via mwdb.cert.pl, accessed on 26 March 2021 malware service, and from the CERT Polska’s internal malware hunting systems. The analyzed pcap files were labeled using Suricata IDS and ET Pro ruleset. The labeling process was performed in multiple steps. Firstly, all pcap files were analyzed using Suricata IDS and these logs were saved. Secondly, alert messages from the IDS logs were reviewed semimanually to include only those related to HTTP requests and the malware family’s name. Based on the SID rule identification number, the alert messages were labeled with the corresponding malware family name using the information from the corresponding IDS rule. Thirdly, all HTTP requests belonging to a particular network flow, for which the reviewed IDS alert existed, were labeled with corresponding alerts. This step was performed with the assumption that all requests within such a network flow should be treated as malicious. Network flows were identified by source and destination IP addresses and ports. Note that in many cases, HTTP requests were labeled with multiple IDS alerts. As the last step, malware requests were labeled with the malware family name. Again this process was performed semiautomatically by reviewing the names of families corresponding to alerts of particular requests. Requests with multiple different family names were analyzed manually. In most cases, it involved aliases of malware, when names were merged to one, or forks of malware families. These were merged to one name or a specific fork name was chosen. For example, all Ursnif family forks were merged, because alert messages were written by different rule authors, thus incorporating inconsistencies in naming, and the provided fingerprints were identical.

The two data sets mentioned above were merged based on the labeled malware family name. They originate nearly from the same source of malware traffic and use the same intelligence source for labeling, mainly ET Pro rulesets. The final malware data set used in further analyses covers 121 popular malware families with 401,566 HTTP requests. The complete data set provides more data; however, only those malware families were chosen that have at least 20 requests. The top 10 malware families sorted by the number of HTTP requests are presented in Table 2, while the complete list is presented in Appendix C.

### 4.2. Data Set of Benign Application

Apart from the malicious data set, the benign one was also necessary. To obtain it, network traffic of benign applications was collected from two sources: (i) popular web browsers, including background traffic, and (ii) popular benign applications running on Windows 10.

#### 4.2.1. Popular Web Browsers

The data set of popular web browsers’ network traffic was generated by the authors in their previous research [17], where it is described in detail. Various web browsers under the control of different versions of the Windows OS were used to visit websites from the list of 500 most popular websites worldwide, extracted from Alexa top 1 million websites worldwide (http://s3.amazonaws.com/alexa-static/top-1m.csv.zip, accessed on 9 February 2017). The websites were accessed between 9 and 15 February 2017 and between 13 and 18 October 2017, depending on the browser. Table 3 contains information about the networking environment and the number of requests observed in each web browser traffic. Including background traffic, this part of the data set contains 194,940 HTTP requests.

#### 4.2.2. Network Traffic of Popular Benign Applications Running on Windows 10

Network traffic of popular benign applications running on Windows 10 was obtained using an experimental environment equipped to perform a man-in-the-middle (MitM) attack on HTTPS network traffic. The main objective was to create a data set of network traffic that would be highly similar to the traffic observed in a home or a small business network.

The experimental environment consisted of two virtualized hosts: one, in the remainder of this section called analysis host, was running Windows 10, and the second one, called *MitM host*, was used to provide Internet connectivity and network traffic dumping. Windows 10 OS was obtained from https://modern.ie, accessed on 26 March 2021 in version 1809. Additional root X.509 certificate was installed in the system to provide means for the MitM mechanism. *MitM host* was based on Ubuntu 20.04 LTS OS, equipped with sslsplit tool to perform a man-in-the-middle attack on HTTPS traffic. All network traffic was routed through the *MitM host*. The experiment was divided into three parts that were executed during six consecutive days. The network traffic was not deciphered during the first part, mainly giving unmangled situation and normal traffic. All OS updates were performed during this period.

In the second part of the experiment, the MitM mechanism was enabled and network traffic on ports 80 and 443 was forwarded through sslsplit. sslsplit was configured to work with the least offensive mode to minimize its impact on the network traffic. The traffic was dumped to pcap files for later analysis. During this period, popular benign applications were installed and run. This includes VLC media player, Adobe Acrobat Reader, Steam, Spotify, Discord, Libre Office, and Microsoft Office. The complete list is available in Appendix B. The applications were used to mimic the behavior of a standard user: creating files with Microsoft Office/LibreOffice suites, saving them to OneDrive cloud repositories, opening some saved files, using e-mail clients to download and send messages, listening to music, or downloading files. In all applications, update modules were used to download any available updates. Additionally, some well-known websites were visited using Google Chrome and Microsoft Edge based on Chromium, including registering and logging on popular social media sites such as Facebook, Instagram, and Outlook.com, accessed on 26 March 2021. Internal Windows applications were also used, including weather, calendar, and movie services. Usage of the MitM during this part of the experiment caused some essential OS services to stop working, including Windows Update and Windows App Store. According to multiple sources [20,21], these services send telemetry data using HTTPS with internal, additional X.509 certificate repository and certificate pinning mechanism. When sslsplit was enabled, these applications encountered the error 80245006.

In the third part, the sslsplit was disabled and traffic was dumped in the same manner as during the first part. This phase provided an environment not impacted by the MitM mechanism, with all OS services operating normally and background services of previously installed applications.

The data set contains 53,717 HTTP requests. The top 10 values of the *User-Agent* header value ordered by the number of requests are presented in Table 4. Note that 2.26% of requests do not contain *User-Agent* header or its value is empty.

### 4.3. Fingerprinting Tools Used for Comparison

Three HTTP fingerprinting tools were used for comparison with Hfinger: FATT, p0f, and Mercury. Their overview is presented in Section 2.2. Their source code versions are presented in Appendix A. Code changes and configuration of the tools used in the analysis are described below.

FATT is used in the version provided by its GitHub repository (https://github.com/0x4D31/fatt/, accessed on 26 March 2021). Additionally, to provide similar test conditions between all tested tools, two types of FATT output are further analyzed. The first one is the header hash as provided by the tool. The second one is the header hash with the value of the *User-Agent* header that is the output of the default reporting mode when used with the command-line interface.

p0f was analyzed using source code parts of its Python port (https://github.com/FlUxIuS/p0f3plus, accessed on 26 March 2021). As the tool fingerprints only *GET* requests, the code was patched to analyze all request types to provide the same base for comparison with Hfinger. The code was also patched to support requests with a nonstandard end of line tag: *LF* instead of *CRLF*.

For analysis of Mercury its Python version *pmercury* was used. Mercury’s analysis process can be modified using a configuration file to manipulate, for example, the list of analyzed headers or the list of headers that should be represented with their values. Thus, for comparison with other tools, two configurations were used: (i) the default, provided by the authors of the tool and (ii) the same as the default but extended with representing the value of the *User-Agent* header in the fingerprint. The source code was patched to support the analysis of nonstandard requests. These were present when analyzing requests with the nonstandard end of line tag: *LF* instead of *CRLF* and those without protocol version definition. Even though such requests are rarely observed in malware traffic, they should be properly handled.

### 4.4. Comparison Measures and Methodology

In this research, the performed analyses and comparisons are based on four measures that, in our opinion, provide useful insights into real-life applications of malware HTTP traffic fingerprinting tools. This includes, for example, the uniqueness of the fingerprint across malware families.

Please note that to minimize the effect of different sizes of request sets of analyzed malware families, the measures are computed as averages of each family’s partial value, not a global value. Firstly, the analyzed phenomena occurrences are counted separately for each family and then the average value is computed and provided as the final measure. With such an approach, all requests in each malware family set can be analyzed, which could not be achieved if, for example, undersampling methods of data set balancing were used. If the measures were counted with a global approach, the families with a significantly higher number of requests (e.g., Dridex, Upatre, or Chthonic) would bias measure’s value impacting the whole analysis.

The comparison measures utilized in this research include: malware collision level, fingerprint generation level, level of collision with benign applications, and entropy. All of them are explained in detail below.

*Malware collision level* provides information on whether any collisions of request fingerprints between malware families occur, that is, whether request fingerprints are seen across multiple families and are not unique to one family. This measure should be as low as possible to provide exclusive and reliable fingerprints. Malware collision level is computed by firstly counting the ratio of requests with fingerprint collision to all requests for each family, then counting the mean value of these ratios across all families. Malware collision level is expressed by Equation (1), where *N* is the number of malware families, $r_{i}^{c}$ denotes the number of requests with fingerprint collision for malware family *i*, and $r_{i}$ expresses the number of all requests for malware family *i*.

(1) $$C_{M}=\frac{\sum_{i=1}^{N}\frac{r_{i}^{c}}{r_{i}}}{N}$$

*Fingerprint generation level* provides information about the number of fingerprints generated for a particular malware family set of requests. It can be interpreted as a measure of an average number of fingerprints generated per analyzed request set (for example, in a single pcap file) but also a measure of the degree to which requests are grouped together. Thus indirectly informing about the degree of a fingerprinting tool’s request information generalization. This measure should be as low as possible but still capable of discerning requests that are actually different. It results from requirements that fingerprinter should extract only necessary information from requests and minimize the number of produced fingerprints, not to overwhelm logging and analytic systems. The measure is computed by counting the average ratio of request fingerprints to all malware families’ requests. Fingerprint generation level is calculated using Equation (2), where *N* is the number of malware families, $f_{i}$ is the number of fingerprints for malware family *i*, and $r_{i}$ is the number of all requests for a malware family *i*.

(2) $$G=\frac{\sum_{i=1}^{N}\frac{f_{i}}{r_{i}}}{N}$$

Note that a trade-off between malware collision and fingerprint generation levels exists. When the fingerprinter extracts more information from requests to provide a more unique set of fingerprints, it decreases the collision level. However, it also provides a larger number of fingerprints, as a result increasing the fingerprint generation level. This trade-off is further analyzed in Section 5.1, where optimization of these measures is performed.

The third measure is the level of *collision with benign applications* that provides information about the number of malware fingerprint collisions with some popular, benign applications. In real-life environments, malware operates along with standard, nonmalicious applications. A good fingerprinter should be capable of producing unique fingerprints both to malware and benign applications, thus providing means for discerning these types of applications. This measure is computed similarly to *malware collision level*, i.e., an average value of the ratio between malware requests with fingerprint collision with benign applications and the number of all requests. The level of collision with benign applications is expressed by Equation (3), where *N* is the number of malware families, $r_{i}^{bc}$ expresses the number of requests with fingerprint collision with benign applications for malware family *i*, and $r_{i}$ denotes the number of all requests for the malware family *i*.

(3) $$C_{B}=\frac{\sum_{i=1}^{N}\frac{r_{i}^{bc}}{r_{i}}}{N}$$

The final, fourth measure provides information about *entropy* of the tool. It is represented as an average Shannon entropy of fingerprints across analyzed malware families represented in bits. Equation (4) provides the formula of this measure, where *N* is the number of malware families and $H_{i}$ Shannon entropy for malware family *i*.

(4) $$E=\frac{\sum_{i=1}^{N}H_{i}}{N}$$

Shannon entropy $H_{i}$ of fingerprints of a particular malware family *i* is defined by Equation (5), where $M_{i}$ denotes the number of fingerprints produced by the tool for the malware family *i*, p($f_{j}$) represents the occurrence probability of fingerprint $f_{j}$ (computed as the number of occurrences of requests with a particular fingerprint *j* divided by the number of all requests of the particular family), and $log_{2}$ is a logarithm with base 2.

(5) $$H_{i}=\sum_{j=1}^{M_{i}}p\left(f_{j}\right)log_{2}\left(p\left(f_{j}\right)\right)$$

Fingerprint entropy *E* can be interpreted as a measure of the average amount of information provided by malware fingerprints of a particular tool. The higher the value, the better, as in this case, fingerprints are more informative.

## 5. Experimental Results

Below we present the experimental evaluation of the proposed Hfinger tool. First of all, we demonstrate how the optimal feature set selection has been performed. Then, we outline the results of the comparison of Hfinger with other existing HTTP fingerprinting tools. Note that all tools were analyzed using fingerprint exact match search, and no fuzzy search mechanisms were utilized, even if the tool under evaluation supports it.

Data sets presented in Section 4 were divided randomly into two equal parts based on the malware family (malware data set) or the application name present in the *User-Agent* string (benign data set). For each malware family/application, 50% of the requests were assigned to the first part used to select the optimal feature set, while the rest of the requests were assigned to the part used for the final evaluation of fingerprinting tools.

### 5.1. Selecting the Optimal Feature Set

The goal of the selection of the optimal feature set is to provide a list of features from those presented in Section 3.2 that will provide the optimal results of the four measures defined in Section 4.4 (i.e., malware collision level, fingerprint generation level, level of collisions with benign applications, and fingerprint entropy). Additionally, some numerical features can be presented with different rounding: with or without fractional component, in the remainder of the text described as a float or as an integer, respectively. Thus, this process will provide information on which rounding would be best for each feature.

The process of feature set selection is based on two steps. Firstly, the defined measures are computed for all 186,623 subsets of features. Secondly, the actual selection was performed using different methods described further in the text, including multiobjective optimization techniques, with the results from the first step.

**Figure 3:**
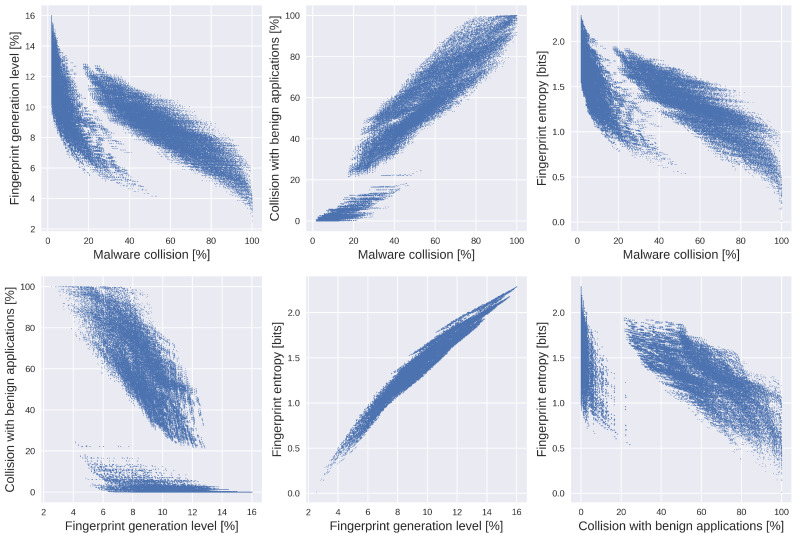
Relationships between defined measures for all possible combinations of feature subsets using training data set. From left to right upper row: (**a**) fingerprint generation level in function of malware collision level, (**b**) level of collision with benign applications in function of malware collision level, (**c**) fingerprint entropy in function of malware collision level. From left to right lower row: (**d**) Level of collision with benign applications in function of fingerprint generation level, (**e**) Fingerprint entropy in function of fingerprint generation level, (**f**) Fingerprint entropy in function of collision level with benign applications.

Figure 3 presents the relationships between all pairs of defined measures: malware collision level, fingerprint generation level, level of collisions with benign applications, and fingerprint entropy for all possible combinations of feature sets.

Analysis of the relationship diagrams in Figure 3 suggests that for many feature sets, with the increase of malware collision levels, the level of collisions with benign applications also increases, while the fingerprint generation level and fingerprint entropy decrease. This follows the intuition that with the increase of fingerprint information, fewer malware requests are incorrectly tagged with the exact representation but for the price of an elevated number of fingerprints.

Based on Figure 3 an interesting observation can also be made. Two distinct result groups can be seen for all diagrams except for fingerprint generation level and fingerprint entropy relationship. One of the groups represents results better suited for optimization. Analysis of feature sets showed that this group contains sets with the order of headers or popular headers and their values, thus indicating a significant impact of those two features on results.

Using results described above, five feature sets (A–E) are selected, and they are presented in Table 5.

The feature sets (A–E) were selected using the following methods. The descriptions include a short explanation of the main motive behind each selection method:

A lexicographic method from multiobjective optimization techniques (see [22]), where firstly malware collision level was minimized, then using this minimal value, the minimal value of fingerprint generation level was selected. Obtained feature sets had equal values of collision level with benign applications and fingerprint entropy. Finally, a feature set with the lowest number of features was chosen. This set has been chosen using a proven method of multiobjective optimization.
B from all feature sets with maximal feature number, the set with the minimal level of fingerprint generation level was selected. One such set existed. This set has been chosen to provide information about all proposed request features but also to minimize number of generated fingerprints. In some analysis scenarios, e.g., highly similar malware, such complete information might be crucial for discerning malware families.
C firstly, feature sets were limited to those containing features from the defined list. The list was compiled based on the authors’ experience with malware analysis and how commonly such features are used in their operational work. The list is formed by features: length of the URI, the extension of the file requested in the URI, representation of header order or representation of popular header’s values (at least one feature from this pair), request method, protocol version, payload length, and Shannon entropy of payload. Secondly, the feature sets were filtered to provide only those with a null level of collisions with benign applications and a minimal level of collisions with malware. The feature set with a higher number of features was chosen from two sets with an identical value of fingerprint generation level and fingerprint entropy.
D firstly, fingerprint generation level was limited to 6% (approximately half of the value observed for the sets chosen with the lexicographic method). Then, feature sets with the lowest number of malware collisions were chosen. From four such sets, one with the lowest number of features was chosen. This set has been chosen to provide significantly lower fingerprint generation levels than other Hfinger’s feature sets that also are comparable to other tools.
E firstly feature sets with the maximum level of fingerprint entropy were chosen, and then feature sets with minimal fingerprint generation level were chosen. Four feature sets were obtained with this method, where malware collision level and level of collision with benign applications were equal. Hence, the set with the highest number of features was chosen. This set has been chosen to provide the highest entropy level but with the minimal possible number of generated fingerprints, thus giving the most informative fingerprints from all feature sets.

The results for these five feature sets, along with the results for other analyzed, existing tools, are presented in Table 6.

### 5.2. Comparison of Hfinger to Other Existing Tools

A final comparison of the results was performed using the remaining 50% of the data set, as described in Section 5. The results for the four defined measures are presented in Table 7.

Based on the results presented in Table 7, a general observation can be made that with the default configuration, Mercury provides the worst levels of collision (both malware and benign applications) and fingerprint entropy for all analyzed tools, 63.34%, 31.95%, and 0.46 bits, respectively. It is followed by FATT with the default configuration with malware collision level at 53.45%, benign application collision level at 25.11%, and fingerprint entropy at 0.51 bits. Nevertheless, these two tools provide the lowest fingerprint generation levels: 3.79% for Mercury and 3.83% for FATT. p0f compared to these two tools results in a lower level of malware and benign applications collisions (15.25% and 10.96% respectively) and a higher level of fingerprint entropy (1.98 bits) but at the cost of a higher fingerprint generation level, i.e., 16.41%.

When the *User-Agent* header value is used as a part of a fingerprint for FATT or Mercury, the tools provide lower levels of collisions, both for malware and benign applications but also higher fingerprint entropy. For FATT, the malware collision level decreases by nearly 32 pp (percentage points), from 53.45% to 21.77%, while for Mercury, it decreases by almost 37 pp, from 63.34% to 26.46%. Collisions with benign applications decrease by nearly 13 pp from 25.11% to 12.22% for FATT and by 16 pp from 31.95% to 15.76% for Mercury. An increase in fingerprint entropy value is observed from 0.51 to 0.87 bits for FATT and from 0.46 to 0.84 bits for Mercury. These improvements of the three measures’ values come with the worsening of the fingerprint generation level that nearly doubles both for FATT (from 3.83% to 6.32%) and Mercury (from 3.79% to 6.27%). These results support intuition of the relationship between the collision level and the fingerprint generation level, i.e., if the tool better discerns applications, the number of fingerprints it provides also elevates.

Further analysis of Table 7 shows that, except feature set D, all other feature sets of Hfinger provide significantly lower levels of malware collision than other tools. Feature sets A, C, and E achieve levels lower than 2% and feature set B lower than 4%. These levels are lower by nearly 60 pp (30 times), compared to the worst value for Mercury, and at least 11 pp (4 times) when compared to the best value of the p0f tool. These feature sets also provide a null value of collisions with benign applications that is lower by 10 to 30 pp when compared to other tools. Hfinger’s feature sets A, B, and C achieve higher fingerprint entropy levels than Mercury and FATT, i.e., 1.72, 1.58, and 1.78 bits, respectively. It is about one bit higher than these two tools. However, only feature set E that was chosen to provide the maximum fingerprint entropy of 2.29 bits achieves a higher level than p0f. As observed before with other tools, Hfinger’s measure improvements increase fingerprint generation level. For feature sets A, B, and C, they are nearly 12% (11.76%, 11.01%, and 12.15% respectively), and for feature set E, it is 15.85%. These values are higher by 5 to 12 pp (2–3 times) compared to FATT and Mercury but lower by 5 pp or nearly equal compared to p0f.

Hfinger’s feature set D was chosen with a focus on providing a lower fingerprint generation level than other feature sets, more comparable to FATT and Mercury; thus, it will be analyzed separately for the sake of brevity of the argument. As Table 7 presents, feature set D achieves lower collision levels for both malware and benign applications when compared to FATT and Mercury. Differences appear in comparing feature set D with the default and nondefault configurations of these two tools. Default configurations achieve lower fingerprint generation levels by nearly 2 pp compared to feature set D but almost the same level for nondefault configurations. The same is observed for fingerprint entropy: default versions achieve lower levels, while nondefault achieve nearly the same values as Hfinger with feature set D. When compared to p0f, feature set D achieves 1.5 pp higher malware collision level, but 10 pp lower fingerprint generation level. The level of collisions with benign software is also lower for feature set D by 9 pp. However, p0f achieves a higher fingerprint entropy level: 1.98 bits when the feature set D: 0.85 bits. The additional perspective of the results for feature set D is provided by the fact that this feature set does not contain information about the values of *User-Agent* header, unlike other Hfinger’s feature sets and unlike p0f, and FATT’s and Mercury’s nondefault configurations. It can be used as a starting point for future work on analyzing how much the *User-Agent* header’s value can impact the fingerprint and its capabilities to identify applications.

Overall, the analysis results show that in the majority, Hfinger achieves significantly lower levels of malware and benign applications collisions than other analyzed tools. It results in higher fingerprint generation levels compared to FATT and Mercury but still lower than those of p0f. Fingerprint entropy for Hfinger is also higher than that of FATT and Mercury; however, only one feature set achieves a higher level of this measure than p0f. Specifically designed to decrease the fingerprint generation level, feature set D achieves lower levels of collisions with malware and benign applications when compared to FATT and Mercury but with similar or only 2 pp higher fingerprint generation level. It also provides similar or higher fingerprint entropy. This feature set produces 1.5 pp more malware collisions than p0f and achieves lower fingerprint entropy and has lower levels of fingerprint generation and collisions with benign applications.

Regarding the above analysis, feature set C has been chosen as a default reporting mode for the Hfinger. Firstly, it provides a similar malware collision level as sets A and E, lower than sets B and D. Secondly, its fingerprint generation level is similar to that of set A and lower than of set E. Thirdly, its fingerprint entropy level is lower than of set E but almost identical of set A. Lastly, feature set C provides information about a higher number of features than set A, giving a more complex overview of a request for analyst. Feature set E achieves this with a higher fingerprint generation level.

## 6. Practical Usage Scenarios and Limitations

In this section practical usage scenarios for Hfinger are presented along with the discussion on limitations of this tool.

### 6.1. Practical Usage Scenarios

Hfinger was designed to be used as a standard network fingerprinting tool, and its usage cases are no different from other tools. It is capable of reading pcap files, thus it can analyze network traffic originating from different sources, for example, malware sandbox systems, honeypots but also enterprise networks. Hfinger can be used directly to analyze network traffic or it can be used as a subsystem, whose output is ingested by other analysis systems. When used as a standalone tool, it can help the analyst in network forensic objectives. While used as a subsystem, it can feed data into network monitoring or event logging systems, for example, SIEM (Security Information and Event Management) solutions.

Fingerprints created by Hfinger can also be used to identify and track malware in different scenarios. For example, when analyzing the network traffic of unknown malware, fingerprints created by Hfinger can be used to identify requests that were previously labeled as belonging to a particular malware family. Moreover, if the analyzed network traffic consists of multiple HTTP requests, fingerprints can help in grouping them, giving a basis for further analysis of the purpose of the requests—whether it was a connectivity check, C&C server check-in, or some other malicious activity. Additionally, Hfinger can extend and complement IDS systems by using alerts to search for requests that were not reported but have the same fingerprint as those identified. Another application is to identify similarities between different malware families when similar fingerprints for both are discovered; however, this can be achieved when using Hfinger with techniques other than default exact match search. These were not analyzed in this paper but can be used as a starting point for future work.

Hfinger cannot analyze HTTP network traffic secured with HTTPS protocol on its own. However, in many environments and architectures, HTTPS traffic can be inspected, for example, by using TLS keys in sandbox systems or TLS inspection systems in corporate networks.

### 6.2. Limitations

The presented research and the proposed tool, apart from the auspicious results as outlined in the previous sections, have their limitations that will be discussed below.

Firstly, the authors put maximum effort into the correct preparation of the data sets; however, not all biases could be eliminated. The malware labeling process involved the usage of the ET Pro IDS ruleset. It is a well-known, industry-tested intelligence source that both false positive and false negative errors could be present. That is, some benign HTTP requests were marked as malware, some malicious requests were not alerted, or the malware name provided by the rule was incorrect. Additionally, although malware families were carefully selected for the analysis, their distribution in terms of malware types might not reflect the actual distribution. These biases could influence the results of the analysis and Hfinger feature set selection process.

Secondly, Hfinger capabilities to analyze many features, including header values, can be less efficient for malware families that introduce many changes in the request structure. This can happen, for example, with malware used to perform DDoS attacks, where it is a common technique to change the value of *User-Agent* header with each request. In such a situation, the number of fingerprints created by Hfinger can increase. However, thanks to the fingerprint’s modular structure, this issue can be addressed by ignoring the part of the fingerprint generating the higher level of noise.

Thirdly, some malware families tend to incorporate mimicking mechanisms to become similar to benign applications. Depending on the level of mimicry, Hfinger can help to uncover it. If the changes are simple, for example, altering the value of the *User-Agent* header to a benign one, the generated fingerprints will show only a change in this value. However, when the whole structure of a request is changed, then the issue is becoming harder to address. In the worst-case scenario, the request can be changed to such a degree that the malware fingerprint can be the same as of a benign application. Nevertheless, we believe that applying such a degree of mimicry mechanisms would require a lot of design effort and, from our experience, is not typical for malware developers.

Finally, fingerprints produced by Hfinger were designed to be used in exact match searches. Potentially, they can also be used to perform fuzzy searching, for example, by using only some parts of the basic fingerprint. However, we considered it is out of the scope of this paper. The main focus during Hfinger design was put on exact matching, which is supported by many security monitoring and logging tools, contrary to fuzzy search. Furthermore, fuzzy search functionality can be provided with different mechanisms, depending on the monitoring system, thus creating problems with interoperability and potential lack of support of some operations. These issues were analyzed during the design phase of Hfinger, and the decision was made to develop a solution that can be easily integrated into existing deployments of various systems and tools. Nevertheless, fuzzy searching or request clustering mechanisms can be treated as our future work.

## 7. Conclusions

This paper presents Hfinger, an HTTP request fingerprinting tool. Hfinger analyzes the network traffic and extracts information from different parts of the HTTP requests to provide a simple and interpretable for analyst representation of requests. The fingerprints provided by the tool can be used for exact match searches to identify similar requests between different pcap files and, as such, aid in threat hunting or as a step to identify unknown malware.

The results presented in this paper show that in the default Hfinger reporting mode, the generated fingerprints are 8–34 times more unique between malware families than in other three similar, community-proven, existing fingerprinting tools: FATT, Mercury, and p0f. In the default reporting mode, Hfinger introduces no collisions between malware and benign applications, contrary to the other tools. The number of generated fingerprints is at most about three times higher when compared to FATT and Mercury but 35% lower compared to p0f. Hfinger achieves higher levels of fingerprint entropy than FATT and Mercury but only slightly lower than p0f. In the authors’ opinion, the three-fold increase in the number of fingerprints is justifiable by the significant (8–34 fold) increase of fingerprint uniqueness. Thus, this analysis confirms that Hfinger is an effective tool for malware HTTP request fingerprinting.

Hfinger can also operate in other reporting modes that can help achieve better fingerprint entropy levels, provide a lower number of fingerprints, or produce information about a broader set of request features. They offer better or at least comparable results for all measures defined in this paper compared to the other analyzed tools.

Future work will focus on enabling fuzzy searching. This includes, for example, capabilities for searching similar requests on the base of a fingerprint’s substring, using a wildcard search or searching depending on the importance of fingerprint elements. Another direction is to use Hfinger as a basis for request clustering mechanisms, which can help to uncover new relations between requests.

## Figures and Tables

**Table 1:** Classification of the reviewed fingerprinting research solutions based on their application scenario.

Usage of Fingerprinting	Examples of Research Solution
Client application or user identification	Laperdrix et al. [6], Segal et al. [15]
Detection of unknown applications	Bortolameotti et al. [7,8]
Service or server identification	Shbair et al. [9], Kim et al. [13]
Malware family identification	Stringhini et al. [10], Bazydło et al. [11]
Malware detection	Blaise et al. [14]
Attack detection	Fachkha et al. [12]
Generic protocol fingerprinting	Holland et al. [16]

**Table 2:** Top 10 malware families by the number of HTTP requests in the final data set.

Malware Family Name	Number of Requests	Percentage of All Requests [%]
Upatre	62,257	15.50
Simda	57,730	14.38
Locky	44,498	11.08
Dridex	30,070	7.49
Arkei	22,057	5.49
DirtJumper	18,486	4.60
Chthonic	14,410	3.59
Vflooder	14,252	3.55
Ursnif	11,756	2.93
Arid Viper APT	10,063	2.51

**Table 3:** Networking environments in which web browser HTTP traffic was analyzed.

Browser Name	Operating System	Number of Requests
Microsoft Edge	Windows 10	17,659
Google Chrome	Windows 7	30,281
Mozilla Firefox (Adobe Flash Player installed)	Windows 7	19,523
Mozilla Firefox	Windows 7	26,131
Microsoft Internet Explorer 11	Windows 7	29,216
Google Chrome	Windows 8.1	22,133
Mozilla Firefox	Windows 8.1	19,082
Microsoft Internet Explorer 11	Windows 8.1	19,807

**Table 4:** The top 10 values of *User-Agent* header value ordered by the number of requests in the data set of network traffic of popular benign applications running on Windows 10.

*User-Agent* Header Value	Percentage of All Requests in the Data Set [%]
Mozilla/5.0 (Windows NT 10.0; Win64; x64) AppleWebKit/537.36 (KHTML, like Gecko) Chrome/83.0.4103.116 Safari/537.36 Edg/83.0.478.58	35.87
Microsoft-Delivery-Optimization/10.0	10.57
Mozilla/5.0 (Windows NT 10.0; Win64; x64) AppleWebKit/537.36 (KHTML, like Gecko) Chrome/83.0.4103.116 Safari/537.36 Edg/83.0.478.61	9.46
Mozilla/5.0 (Windows NT 10.0; Win64; x64) AppleWebKit/537.36 (KHTML, like Gecko) Chrome/83.0.4103.116 Safari/537.36	6.50
Mozilla/5.0 (Windows NT 10.0; Win64; x64) AppleWebKit/537.36 (KHTML, like Gecko) Chrome/64.0.3282.140 Safari/537.36 Edge/18.17763	5.27
Mozilla/5.0 (Windows; U; Windows NT 10.0; en-US; Valve Steam Client/default/1591251555; ) AppleWebKit/537.36 (KHTML, like Gecko) Chrome/79.0.3945.117 Safari/537.36	2.82
Mozilla/5.0 (Windows NT 10.0.17763; Win64; x64) AppleWebKit/537.36 (KHTML, like Gecko) Slack/4.7.0 Chrome/83.0.4103.119 Electron/9.0.5 Safari/537.36 Sonic Slack_SSB/4.7.0	2.52
Valve/Steam HTTP Client 1.0 (0)	1.65
microsoft.windowscommunicationsapps	1.51
Microsoft Office/16.0 (Windows NT 10.0; Microsoft Outlook 16.0.13001; Pro)	1.45

**Table 5:** Selected feature sets.

Feature Set Name	Feature List
A	average directory length represented as an integer
	average value length represented as a float
	number of directories
	extension of requested file
	order of headers
	popular headers and their values
	payload length represented as a float
B	average directory length represented as an integer
	average value length represented as an integer
	number of directories
	extension of requested file
	URI length represented as an integer
	variable length represented as an integer
	number of variables
	request method
	version of protocol
	order of headers
	popular headers and their values
	presence of non-ASCII characters
	payload entropy represented as an integer
	payload length represented as an integer
C	average directory length represented as an integer
	average value length represented as a float
	number of directories
	extension of requested file
	URI length represented as an integer
	request method
	version of protocol
	order of headers
	popular headers and their values
	presence of non-ASCII characters
	payload entropy represented as an integer
	payload length represented as a float
D	average directory length represented as an integer
	average value length represented as an integer
	extension of requested file
	URI length represented as an integer
	order of headers
E	average directory length represented as a float
	average value length represented as a float
	number of directories
	extension of requested file
	URI length represented as a float
	variable length represented as a float
	request method
	version of protocol
	order of headers
	popular headers and their values
	presence of non-ASCII characters
	payload entropy represented as a float
	payload length represented as a float

**Table 6:** Optimization results for five selected feature sets compared to other analyzed tools. The *UA* suffix marks nondefault configuration of tools supporting *User-Agent* header value as a part of the fingerprint.

Tool	Malware Collision Level [%]	Fingerprint Generation Level [%]	Level of Collisions with Benign Applications [%]	Fingerprint Entropy [bits]
Hfinger (A)	1.76	11.76	0.00	1.72
Hfinger (B)	3.49	11.19	0.00	1.57
Hfinger (C)	1.76	12.09	0.00	1.77
Hfinger (D)	16.85	5.95	1.11	0.87
Hfinger (E)	1.76	15.96	0.00	2.29
FATT	53.04	4.16	24.45	0.54
FATT UA	22.11	6.63	11.87	0.88
Mercury	64.15	4.11	31.33	0.49
Mercury UA	27.13	6.58	15.26	0.85
p0f	15.70	16.71	11.25	1.99

**Table 7:** Final evaluation of Hfinger’s five selected feature sets compared to other analyzed tools. The *UA* suffix marks nondefault configuration of tools supporting *User-Agent* header value as a part of fingerprint.

Tool	Malware Collision Level [%]	Fingerprint Generation Level [%]	Level of Collisions with Benign Applications [%]	Fingerprint Entropy [bits]
Hfinger (A)	1.85	11.76	0.00	1.72
Hfinger (B)	3.58	11.01	0.00	1.58
Hfinger (C)	1.85	12.15	0.00	1.78
Hfinger (D)	16.78	5.78	1.51	0.85
Hfinger (E)	1.78	15.96	0.00	2.30
FATT	53.45	3.83	25.11	0.51
FATT UA	21.77	6.32	12.22	0.87
Mercury	63.34	3.79	31.95	0.46
Mercury UA	26.46	6.27	15.76	0.84
p0f	15.25	16.41	10.96	1.98

## Data Availability

Publicly available pcap file data set of Malware Capture Facility Project was analyzed in this study. It can be found here: https://www.stratosphereips.org/datasets-malware, accessed on 26 March 2021. The pcap file data set provided by CERT Polska cannot be shared due to legal, confidentiality, and privacy issues.

## Appendix A. List of Source Code Versions of the Analyzed Fingerprinting Tools

**FATT**:

https://github.com/0x4D31/fatt/commit/314cd1ff7873b5a145a51ec4e85f6107828a2c79, accessed on 26 March 2021

**p0f3plus**:

https://github.com/FlUxIuS/p0f3plus/commit/748cc69cc996e830f258f3f3c7b95ca7a4a74a3e, accessed on 26 March 2021

**Mercury**:

https://github.com/cisco/mercury/commit/500f5b74a710c0f1c423b8cb370c667aae44a7e3, accessed on 26 March 2021

## Appendix B. List of Installed Benign Applications Used in Experiments

- Adobe Reader
- Discord
- GIMP
- LibreOffice
- Mozilla Thunderbird
- Notepad++
- Microsoft Office 2019 Home and Business
- Skype
- Slack
- Spotify
- Steam
- VLC media player
- Zoom
- µtorrent

## Appendix C. Malware Families of the Final Malware Data Set Sorted by the Number of HTTP Requests

**Table A1:** Malware families of the final malware data set sorted by the number of HTTP requests in the final data set.

Malware Family Name	Number of Requests	Malware Family Name	Number of Requests
Upatre	62,257	KeyBase	141
Simda	57,730	STOP	139
Locky	44,498	Nessfi	136
Dridex	30,070	Jaff	136
Arkei	22,057	GrayBird	136
DirtJumper	18,486	Cannibal	130
Chthonic	14,410	1ms0rry	129
Vflooder	14,252	IcedID	122
Ursnif	11,756	Wannacry	113
Arid Viper APT	10,063	Adylkuzz	111
Emotet	9662	Amadey	103
Nemucod	8857	ArtraDownloader	99
Houdini	7583	Zeprox	96
Miuref/Boaxxe	7501	PowershellEmpire	88
Pushdo.S	7012	MegalodonHTTP	88
SmokeLoader	6523	BlackshadesRAT	82
Andromeda	5839	Banload	80
Nymaim	5590	GrandSteal	76
Matsnu	5522	Mokes	73
LokiBot	4415	EightRed	73
Kovter.B	4332	ZeroHTTP	70
Tinba	4004	Sakula	67
Formbook	3496	NetSupport	65
AgentTesla	3052	Legion	62
Gaudox	2880	FindPOS	60
BlackNET	2822	DDI.Bot	59
AZORult	2057	Agent.ZJL	57
Mydoom	1833	Adware.Liuliangbao.A	55
Htbot	1730	DCRS	54
Neutrino	1697	Dalexis	52
Kronos	1692	FTCode	50
PUP.Linkury	1481	MSIL.adv	47
Trickbot	1255	Maze	46
Necurs	1158	KPOT	45
Sage	1145	Sality	41
Hancitor	1034	Madness	41
CryptoWall	613	Dimnie	38
Pony	607	Instagram Like Bot	37
Wizzcaster	567	H1N1	36
QuantLoader	538	Panda	35
TVRat	436	Ratankba	34
Kelihos.F	406	Zeroaccess	33
MedusaHTTP	403	DownloadGuide	33
Karmen	397	Betabot	31
GuLoader	383	Alina.POS	31
KINS	351	SocStealer	30
Tofsee.AX	338	Sezin	30
Predator The Thief	286	Scarab	30
InstallCapital	274	Golroted.B	30
Terdot	256	Agima.o	30
TinyNuke	250	CobaltStrike	29
ColorFish	242	Philadelphia	28
HawkEye	234	Dapato	27
Sarwent	229	Mole	26
GandCrab	229	TorrentLocker	24
DustySky	200	FusionCore	23
Phorpiex	190	Qadars	20
DirCrypt	174	KrugBOT	20
Alphacrypt	174	JakyllHyde	20
Donvibs	168	HPDefender.B	20
DiamondFox	153		
